# A Clinical Prediction Rule for Protease Inhibitor Resistance in Patients Failing Second-Line Antiretroviral Therapy

**DOI:** 10.1097/QAI.0000000000001923

**Published:** 2019-03-01

**Authors:** Karen Cohe, Annemie Stewart, Andre P. Kengne, Rory Leisegang, Marla Coetsee, Shavani Maharaj, Liezl Dunn, Michael Hislop, Gert van Zyl, Graeme Meintjes, Gary Maartens

**Affiliations:** * Division of Clinical Pharmacology, Department of Medicine, University of Cape Town, Cape Town, South Africa; † Department of Medicine, University of Cape Town, Cape Town, South Africa; ‡ Non-communicable Diseases Research Unit (NCDRU), South African Medical Research Council, Cape Town, South Africa; § Aid for AIDS Management (Pty) Ltd, Cape Town, South Africa; ∥ Division of Medical Virology, Department of Pathology, NHLS and Stellenbosch University, Stellenbosch, South Africa; ¶ Centre for Infectious Diseases Research in Africa (CIDRI-Africa), Institute of Infectious Disease and Molecular Medicine, University of Cape Town, Cape Town, South Africa

**Keywords:** protease inhibitor, clinical prediction rule, virological failure, genotypic resistance, second-line antiretroviral therapy

## Abstract

**Background:**

Most adults with virological failure on second-line antiretroviral therapy (ART) in resource-limited settings have no major protease inhibitor (PI) resistance mutations. Therefore, empiric switches to third-line ART would waste resources. Genotypic antiretroviral resistance testing (GART) is expensive and has limited availability. A clinical prediction rule (CPR) for PI resistance could rationalize access to GART.

**Setting:**

A private sector ART cohort, South Africa.

**Methods:**

We identified adults with virologic failure on ritonavirboosted lopinavir/atazanavir-based ART and GART. We constructed a multivariate logistic regression model including age, sex, PI duration, short-term adherence (using pharmacy claims), concomitant CYP3A4-inducing drugs, and viral load at time of GART. We selected variables for the CPR using a stepwise approach and internally validated the model by bootstrapping.

**Results:**

148/339 (44%) patients had PI resistance (defined as ≥ 1 major resistance mutation to current PI). The median age was 42 years (interquartile range 36–48), 212 (63%) were females, 308 (91%) were on lopinavir/ritonavir, and median PI duration was 2.6 years (interquartile range 1.6–4.7). Variables associated with PI resistance and included in the CPR were age {adjusted odds ratio (aOR) 1.96 (95% confidence interval [CI]: 1.42 to 2.70) for 10-year increase}, PI duration (aOR 1.14 [95% CI: 1.03 to 1.26] per year), and adherence (aOR 1.22 [95% CI: 1.12 to 1.33] per 10% increase). The CPR model had a c-statistic of 0.738 (95% CI: 0.686 to 0.791).

**Conclusions:**

Older patients with high adherence and prolonged PI exposure are most likely to benefit from GART to guide selection of a third-line ART regimen. Our CPR to select patients for GART requires external validation before implementation.

## INTRODUCTION

Virological failure develops frequently in adults on second-line protease inhibitor (PI)-based antiretroviral therapy (ART) in resource-limited settings, occurring in 38% of patients by 36 months in a systematic review.^1^ However, only a minority of patients failing second-line ART have major PI resistance mutations.^1–8^ Therefore, switching all patients failing second-line ART empirically to a third-line ART regimen, as is done for first-line ART failure, would result in many unnecessary ART regimen switches and would fail to address poor adherence, which is the sole cause of virologic failure in most patients with no PI resistance mutations. Genotypic antiretroviral resistance testing (GART) to detect HIV-1 resistance mutations identifies those patients who require switching to a third-line ART regimen. However, GART is expensive and access is limited in resource-limited settings. Third-line ART regimens are costly. Simple measures to predict PI resistance in patients with virologic failure on second-line ART could be used to rationalize access to GART or, in areas where GART is unavailable, to empirically switch to third-line ART.

We identified predictors of major PI resistance mutations in a cohort of South African patients failing second-line ART. We used these predictors to construct a clinical prediction rule (CPR) to identify patients most like to have major PI resistance mutations.

## METHODS

We identified adult patients on PI-based second-line ART in the Aid for AIDS (AfA) cohort who had GART performed between January 2004 and December 2013. AfA is a large Southern African private sector HIV management program. AfA collects demographic, laboratory, clinical, and medication claims data on individuals registered for HIV benefits. Although AfA is a private sector program, their guidelines are similar to the WHO guidelines^9–11^ with standardized ART regimens (nonnucleoside reverse transcriptase inhibitor-based for first-line and PI-based for second-line) and 6 monthly monitoring of CD4 count and viral load (VL). Patients were covered by private health insurance, and no co-payment was required for ART, VL, and CD4 monitoring, or doctor visits, or for GART (which required preauthorization). Virological failure was defined as 2 consecutive VLs greater than 1000 copies per milliliter.

Inclusion criteria were: ≥18 years old, GART result, taking lopinavir or atazanavir (boosted with ritonavir) second-line ART at the time of GART, and at least 4 months of exposure to the boosted PI. We excluded patients taking a second PI other than ritonavir in addition to atazanavir or lopinavir.

Where patients had more than 1 GART performed on second-line ART, we included only the first result. We calculated duration of PI exposure from PI initiation to date of the first GART. We used the Stanford University HIV Drug Resistance Database (version 6.3.1 released on September 20, 2013; downloaded on October 31, 2013) to identify major PI resistance mutations. We categorized patients as having PI resistance if they had one or more major PI resistance mutations to the PI they were taking at the time of GART. We calculated Stanford resistance score for lopinavir, atazanavir, and darunavir (darunavir is the PI that is commonly used in salvage therapy regimens in South Africa).

We determined ART adherence using pharmacy claims data over 4 months, as we had previously shown that this interval predicted virologic failure on second-line ART.^12^ We calculated the proportion of days where the patient would not have had any medicine, based on the refill intervals for that medicine. Percentage adherence was calculated as 100 minus percentage of days “out of medicine” for the 4-month period.

We constructed a multivariate logistic regression model of associations with major PI resistance. The following variables were included in the multivariate model based on an a priori decision: age, sex, duration of exposure to ritonavir-boosted lopinavir/atazanavir, adherence in 4 months before GART, exposure to concomitant drugs known to induce PI metabolism, and VL at time of GART.

We selected variables for inclusion in the CPR for PI resistance using a stepwise approach as described by Collett.^13^ We internally validated our model by bootstrap resampling-based 2000 replications. The discriminatory performance of the model was assessed with the use of c-statistic and calibration with the use of Hosmer-Lemeshow. To operationalize the model for application in routine setting, we categorized included variables and assigned points to each level of each variable based on the adjusted beta coefficients obtained from the model as described by Sullivan.^14^ The receiver operating characteristic (ROC) curve and Youden’s^15^ index method were then used to derive the optimal cutoff point score. Sensitivity and specificity were estimated to characterize the diagnostic performance of the model at this cutoff and other point scores.

## RESULTS

We included 339 patients in the analysis. Baseline characteristics, stratified by PI resistance, are shown in Table 1. One or more major PI resistance mutations (list of mutations is given in Table 1, Supplemental Digital Content, http://links.lww.com/QAI/B254) were detected on GART in 148/339 (44%) patients. The proportion of patients with PI resistance was similar in the lopinavir and atazanavir groups; 136/308 (44%) and 12/31 (39%), respectively (*P* = 0.560). By Stanford scoring, 76/339 (22%) had high-level resistance to lopinavir, 45/339 (13%) had high-level resistance to atazanavir, and 2/339 (0.6%) had high-level resistance to darunavir. Details of Stanford scores are given in Table 3, Supplemental Digital Content, http://links.lww.com/QAI/B254. Mutations to nucleoside reverse transcriptase inhibitors and/or nonnucleoside reverse transcriptase inhibitors were detected in 227 patients (67%). Details of reverse transcriptase inhibitor mutation detected are given in Table 2, Supplemental Digital Content, http://links.lww.com/QAI/B254.

In the multivariate logistic regression model, the presence of major PI resistance mutations was associated with older age, longer duration of PI exposure, and higher adherence in the 4 months before genotyping (Table 2). There was no association with sex, exposure to PI metabolism–inducing drugs, or VL at time of genotyping (Table 2).

**Figure 1.**
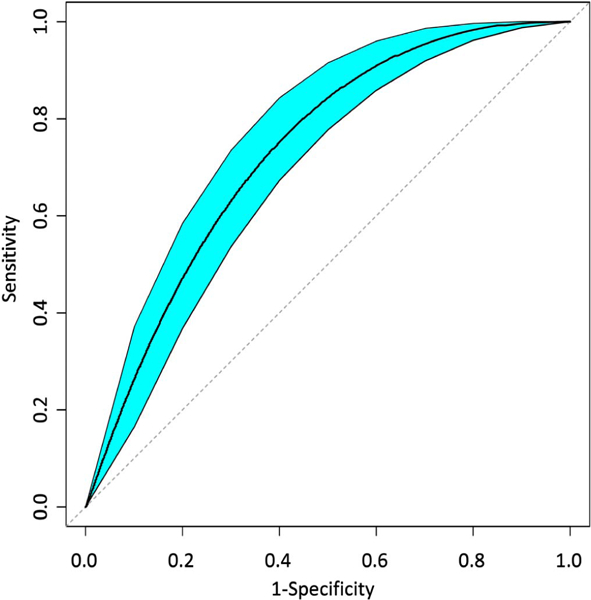
Smooth ROC curve. Shaded area includes the 95% CI derived from the bootstrap, based on 2000 replications. Area under the curve 0.738 (95% CI: 0.686 to 0.791).

Age, duration of PI exposure, and adherence were selected for inclusion in the CPR. The C-statistic for this final model was 0.738 (95% confidence interval [CI]: 0.686 to 0.791) in the derivation sample and 0.736 (percentiles method-based 95% CI: 0.722 to 0.741) in bootstrap internal validation (Fig. 1), with a nonsignificant optimism (0.007 [95% CI: −0.046 to 0.056], *P* = 0.796). The CPR multivariate model had acceptable calibration. The CPR is shown in Table 3. The optimal cut point on the ROC curve corresponded to a score of 8/15, which identifies patients with major PI resistance mutations with 75% sensitivity and 68% specificity (Table 3). However, a score of 6/15 identifies patients with major PI resistance mutations with 94% sensitivity and specificity of 31%, which could be used to rationing to GART where necessary, without missing too many patients with PI resistance.

## DISCUSSION

We found ≥1 major PI resistance mutations in 44% of patients failing second-line ART. Predictors of PI resistance in this cohort were older age, longer exposure to PI-based ART, and higher adherence over the 4 months preceding GART. We developed a CPR, which could be used to identify patients likely to benefit from immediate GART because of high likelihood of PI resistance and those with low likelihood of PI resistance who require enhanced adherence support. A score of 6/15 could be used to ration access to GART because it correctly identifies more than 90% of patients with PI resistance and has reasonable specificity.

The proportion of patients failing second-line ART with PI resistance that we found is higher than previously described in the South African public sector.^2,3,5,6,16^ The high proportion of patients with PI resistance that we observed may in part be due to the prolonged exposure to PIs in this cohort, as the AfA program started providing ART several years before the inception of the South African public sector ART program. In addition, there may be some selection bias, as patients known to be poorly adherent may have been denied preauthorization of GART by AfA. A Nigerian study reported PI resistance in 62% of patients failing PI-based second-line ART with GART being limited to patients with good adherence.^17^ However, a recent Kenyan study found one or more major PI resistance mutations in 32% of unselected patients with second-line ART failure and a median duration on PI-based ART of 3.1 years,^8^ suggesting that PI resistance may become more common in patients with virologic failure on second-line ART in Africa because ART programs mature and there is longer duration of exposure to PIs.

We found a positive association between adherence and PI resistance. Excellent (>95%) adherence is required to protect against the selection of resistance, whereas poor adherence does not provide enough selection pressure for resistance, resulting in a bell-shaped curve for the PI resistance-adherence relationship.^18^ Our finding that older age predicted PI resistance is therefore likely explained by the higher adherence to ART seen with increasing age. Few studies have assessed predictors of PI resistance in patients failing second-line ART in resource-limited settings. In contrast to our findings, a Kenyan study found no association between PI resistance and age or duration of ART, but they only reported on 123 patients with PI resistance in 39; therefore, their study may have lacked sufficient power.^8^

Our study has limitations. First, our findings were from a private sector cohort and may not be generalizable to the South African public sector or to other resource-limited settings. Second, adherence was measured by pharmacy refill only, with no verification that medication was actually taken by patients. Adherence may have been overestimated for patients receiving ART by monthly courier delivery. Third, our ability to distinguish differences in proportions of patients with PI resistance by the PI group was limited by the small sample size in the atazanavir group. Strengths of our study are the prolonged duration of PI exposure in the cohort, the likely accuracy of our model as we had more than double the recommended number of 10 outcomes per candidate variable for the stability of logistic regression models,^19^ and the use of the objective adherence measure of pharmacy refills, which is readily obtainable in most ART programs, in our predictive model.

Based on our analysis, older patients with high adherence and prolonged PI exposure are most likely to benefit from GART to guide selection of a third-line ART regimen. The simple CPR that we developed requires external validation. A validated CPR could be a useful tool to select patients for GART in resource-limited settings.

## Supplementary Material

Supplemental data

## Figures and Tables

**TABLE 1.** Baseline Characteristics of 339 Patients Staratified by the Presence of PI Resistance (Defined as ≥ 1 Major PI Resistance Mutation on GART)

Variable	Category	PI Resistance, n = 148	No PI Resistance, n = 191	*P*	All, n = 339

Age, median yrs (IQR)		44 (40–48)	39 (35–45)	<0.001	42 (36–48)
Sex	Female, n (%)	81 (55)	131 (69)	0.009	212 (63)
	Male, n (%)	67 (45)	60 (31)		127 (37)
CD4 count, median cells/μL (IQR)		114 (39–213)	137 (39–238)	0.404	124 (39–229)
VL, median log_10_ (IQR)		4.9 (4.2–5.4)	4.9 (4.3–5.4)	0.712	4.9 (4.3–5.4)
Concomitant antiretrovirals*	TDF + FTC/3TC, n (%)	72 (49)	84 (44)		156 (46)
	AZT + DDI, n (%)	33 (22)	29 (15)		60 (18)
	AZT + 3TC, n (%)	15 (10)	39 (20)		53 (16)
	Other combinations, n (%)	27 (18)	39 (20)		66 (19)
	PI used				
	Lopinavir, n (%)	136 (92)	172 (90)		308 (91)
	Atazanavir, n (%)	12 (8)	19 (10)	0.560	31 (9)
PI duration, median yrs (IQR)		3.2 (1.9–5.9)	2.2 (1.4–4.0)	<0.001	2.6 (1.6–4.7)
Adherence, median % past 4 mo (IQR)		98.3 (90.0–100)	93.3 (45.8–100)	<0.001	96.7 (73.3–100)
CYP3A4-inducing drugs	Any past 12 mo, n (%)	12 (8)	19 (10)	0.607	31 (9)
	Rifampicin, n (%)	6(4)	14 (7)		20 (6)
	Phenytoin, n (%)	1 (1)	1 (1)		2(1)
	Carbamazepine, n (%)	5(3)	6 (3)		11 (3)

* One patient was on lopinavir-ritonavir monotherapy.

3TC, lamivudine; AZT, azidothymidine; DDI, didanosine; FTC, emtricitabine; IQR, interquartile range.

**TABLE 2.** Univariate and Multivariate Associations With Having One or More Major PI Resistance Mutations

Variable	Category (n)	Crude			Adjusted		
		OR	95% CI	Wald Test P	OR	95% CI	Wald Test P

Age*		2.12	1.57 to 2.86	<0.001	1.96	1.42 to 2.70	<0.001
Sex	f(211)	Referent			Referent		
	m(128)	1.81	1.16 to 2.82	0.009	1.38	0.84 to 2.26	0.204
VL†		0.96	0.74 to 1.25	0.784	1.13	0.84 to 1.52	0.424
PI duration‡		1.18	1.08 to 1.29	<0.001	1.14	1.03 to 1.26	0.009
Adherence§		1.23	1.13 to 1.34	<0.001	1.22	1.12 to 1.33	<0.001
Inducing drugs	No (308)	Referent			Referent		
	Yes (31)	0.80	0.37 to 1.70	0.561	0.85	0.37 to 1.95	0.696

* Per 10-year increment in age.

† Per log_10_ increase in VL at time of genotyping.

‡ Per 1-year increase in duration.

§ Per 10% increase over past 4 months.

OR, odds ratio.

**TABLE 3.** CPR for Major PI Resistance Mutations

	
Risk factor	Points Assigned	Score	Predicted Probability	Sensitivity (%)	Specificity (%)
	
**Age (yrs)** 18–29	0	0–2	≤8.7	100	≤2
30–39	2	3	11.9	99	8
40–49	4	4	16.2	99	12
50–59	6	5	21.6	98	19
60–65	8	6	28.2	94	31
**Duration on PI (yrs)**		7	35.8	82	48
<2 years	0	8	44.3	75	68
2–4 years	1	9	53.1	60	75
5–11 years	3	10	61.7	39	85
**Adherence last 4 mo**		11	69.7	32	91
0–39%	0	12	76.6	5	98
40–60%	2	13	82.4	5	98
60–80%	3	14–15	≥90.4	≤1	100
80–100%	4				
	
